# High-throughput gene expression profiling of memory differentiation in primary human T cells

**DOI:** 10.1186/1471-2172-9-44

**Published:** 2008-08-01

**Authors:** W Nicholas Haining, Jill Angelosanto, Kathleen Brosnahan, Kenneth Ross, Cynthia Hahn, Kate Russell, Linda Drury, Stephanie Norton, Lee Nadler, Kimberly Stegmaier

**Affiliations:** 1 Department of Pediatric Dana-Farber Cancer Institute, 44 Binney Street, Boston, MA 02115, USA; 2 Department of Medical Oncology, Dana-Farber Cancer Institute, 44 Binney Street, Boston, MA 02115, USA; 3 Division of Hematology/Oncology, Children's Hospital Boston, USA; 4 Broad Institute of Harvard and MIT, USA

## Abstract

**Background:**

The differentiation of naive T and B cells into memory lymphocytes is essential for immunity to pathogens. Therapeutic manipulation of this cellular differentiation program could improve vaccine efficacy and the in vitro expansion of memory cells. However, chemical screens to identify compounds that induce memory differentiation have been limited by 1) the lack of reporter-gene or functional assays that can distinguish naive and memory-phenotype T cells at high throughput and 2) a suitable cell-line representative of naive T cells.

**Results:**

Here, we describe a method for gene-expression based screening that allows primary naive and memory-phenotype lymphocytes to be discriminated based on complex genes signatures corresponding to these differentiation states. We used ligation-mediated amplification and a fluorescent, bead-based detection system to quantify simultaneously 55 transcripts representing naive and memory-phenotype signatures in purified populations of human T cells. The use of a multi-gene panel allowed better resolution than any constituent single gene. The method was precise, correlated well with Affymetrix microarray data, and could be easily scaled up for high-throughput.

**Conclusion:**

This method provides a generic solution for high-throughput differentiation screens in primary human T cells where no single-gene or functional assay is available. This screening platform will allow the identification of small molecules, genes or soluble factors that direct memory differentiation in naive human lymphocytes.

## Background

After antigen encounter populations of naive lymphocytes cells differentiate through an effector state into memory cells that confer protective immunity to the host [1,2]. Memory lymphocytes acquire functions during this differentiation process that are necessary for their immunologic efficacy, including a lowered threshold for proliferation and acquisition of effector functions, and the ability to self-renew for the life-time of the host.

Historically, vaccines have been used to induce antigen-specific T and B cell memory and are highly effective against many pathogens [3]. However, diseases like HIV and cancer that are characterized by defective T cell function are refractory to current vaccination approaches [4,5]. The ability to manipulate directly the differentiation of memory lymphocytes would therefore have great importance in improving immunotherapies for cancer and chronic viral diseases. However, the regulatory mechanisms that govern the differentiation of naive lymphocytes into memory cells are not completely understood. This has precluded the development of targeted therapies that could influence the cellular differentiation program underlying memory development [2].

High-throughput small-molecule screens are being increasingly used as a tool to identify compounds that can direct cellular differentiation from a precursor cell into a more differentiated cell-type [6]. This raises the possibility that similar approaches could be used to identify small molecules that induce the full or partial differentiation of naive cells into those that have functional characteristics of memory cells. Small molecules capable of promoting memory differentiation could 1) serve as tool compounds to understand better memory differentiation: and 2) have therapeutic applications in the *ex-vivo* expansion of antigen-specific T cells or as molecularly targeted vaccine adjuvants. Applying this approach to memory differentiation, however, is limited by significant obstacles. Differentiation screens rely on high-throughput assays that can detect the emergence of a more differentiated state. This is often achieved by the use of a reporter gene assay that monitors the expression level of a single differentiation marker gene [7,8]. Reporter gene screens have been used to identify compounds that partially induce either neural or cardiac differentiation in an undifferentiated cell line. However, the memory lymphocyte pool is highly heterogeneous, and no single gene can reliably distinguish naive and memory lymphocytes [1]. Other screening approaches have assayed for a functional read-out such as proliferation to detect compounds that promote a more differentiated state in neural precursor cells [9], but the cardinal functional characteristics of memory cells such as longevity and the ability to self-renew are not easily assayed in vitro.

Genome-wide transcriptional profiling of naive, effector and memory T cells has demonstrated that extensive reprogramming of gene expression occurs during memory differentiation [10,11]. We have previously used cross-species genomic analysis to identify the transcriptional programs that occur during memory differentiation in humans and mice [12]. We demonstrated that these coordinately regulated transcriptional programs, or differentiation signatures, are highly evolutionarily conserved, and shared by multiple lymphoid lineages [12]. This suggests that although no single gene can serve as a marker for memory differentiation, a complex signature of genes may be used to identify populations of cells that have adopted a differentiation state of interest. We therefore sought to develop a robust assay that could discriminate between naive and memory states in primary human lymphocytes based not on a single reporter gene, but on a complex gene expression profile. Previous work in cancer cell lines has demonstrated the utility of using a complex gene expression signature to detect cellular differentiation [13,14]. We therefore tested whether a gene expression-based assay could be used as a high-throughput assay to distinguish primary human naive and memory-phenotype T cells.

## Results

### Detection of differentiation signatures by ligation-mediated amplification

**Figure 1:**
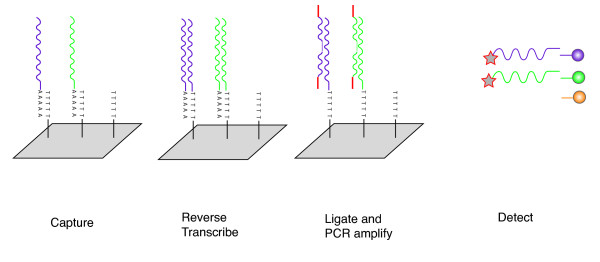
Schema representing steps involved in ligation-mediated amplification.

Ligation-mediated amplification with bead-based detection has been described in detail previously [14-16]. In brief, mRNAs are captured on oligo-dT-coated 384-well plates and reverse transcribed to generate first strand cDNA covalently linked to the plate (Fig. 1). Gene-specific 20-mer oligonucleotides corresponding to the signature genes are then annealed to the cDNA template. The oligos are designed in such a way that primer pairs anneal to adjacent stretches of the gene, allowing the ends of the primer pairs to abut. This is necessary for the ligation step, which will only occur when the abutting ends of the primer pairs are held adjacent to each other by binding the corresponding cDNA. Both gene-specific oligos incorporate a common flanking sequence that allows PCR amplification using a single set of PCR primers that will amplify all gene-specific amplicons. This amplification technique enables the simultaneous amplification of the signature transcripts in a highly reproducible manner that is faithful to the relative abundance of the starting mRNAs. To resolve the relative amounts of each gene-specific amplicon, one partner of each of the oligo pairs incorporates a unique barcode sequence that binds to a fluorescent bead linked to an oligo with a corresponding antisense sequence. Following PCR amplification, individual gene-specific amplicons are distinguished by the fluorescence emission spectra of the barcode-oligo-tagged beads to which they bind. To identify how much amplicon is bound to each bead, one of the PCR primers is biotinylated to allow detection of the relative abundance of the amplicon bound to each bead using streptavidin-phycoerythrin. Up to 100 fluorescently distinct beads are used allowing the quantitation of transcripts from up to 100 genes. The emission spectrum of each bead denotes the identity of the gene; the phycoerythrin intensity the transcript's abundance.

### Selection of Marker Genes to Distinguish Naive and Memory-Phenotype CD4 T cells

**Figure 2:**
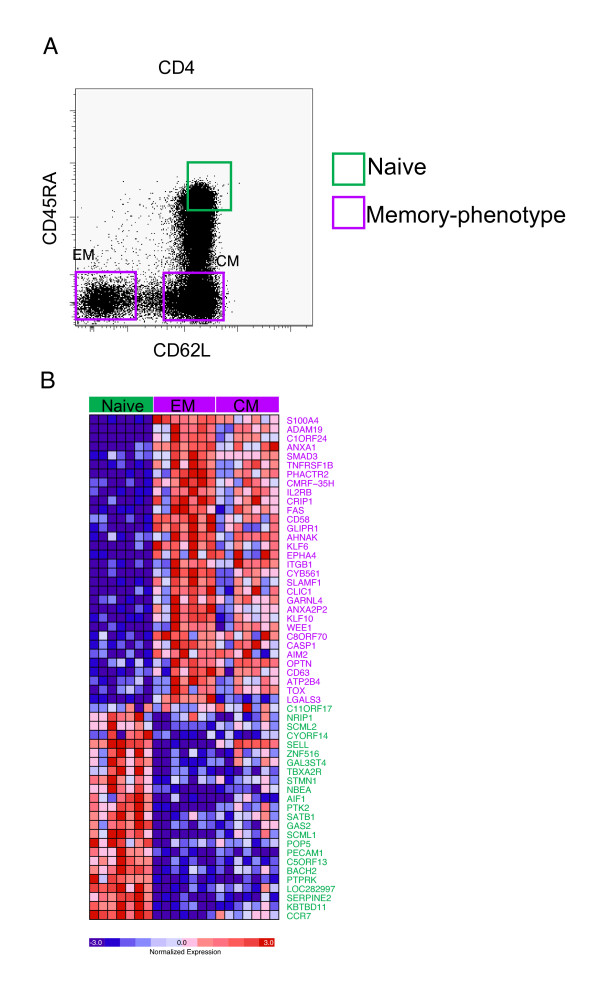
**Gene-expression profiling of naive and memory-phenotype human CD4 T cells**. (A) Peripheral blood T cells, gated on CD4 T cells, stained to identify naive (green gate), central memory (CM) or effector memory (EM) cells. (B) Expression profile of genes differentially expressed in naive and memory-phenotype CD4 T cells sorted using the gates shown in (A). Each row represents an individual gene, and each column a different sample of naive, EM or CM CD4 T cells from seven healthy donors. Genes upregulated in memory-phenotype cells are shown in purple, and those upregulated in naive cells in green.

As proof of principle, we tested whether we could use this approach to distinguish between naive human CD4 cells and memory-phenotype cells on the basis of a well-defined differentiation signature. To identify a differentiation signature that was characteristic of the memory state, we analyzed our previously-published gene expression profiles of human and mouse memory lymphocytes [12]. In a two-step analytic process, we first identified a set of genes differentially expressed by human memory-phenotype CD4 T cells compared to their naive precursors (Fig 2.). Then, we refined this list using gene-set enrichment analysis to identify only those genes present in the human signature that were also upregulated during the differentiation of mouse memory CD8 T cells as we have done previously [12]. This "filtering" step through a gold-standard model of functional memory lymphocytes allowed us to focus on a robust signature of genes characteristic of the memory, rather than memory-phenotype, state. From this list we selected genes that showed the highest signal-to-noise ratio in the microarray analysis of naive and memory-phenotype CD4 T cells (Fig 2). Genes were selected to include those upregulated in memory-phenotype CD4 T cells compared to naive cells (Fig 2B, purple text) and those downregulated in memory-phenotype cells compared to naive cells (Fig 2B, green text). The selection of genes that were differentially expressed in both directions served to reduce the chance of detecting compounds that caused indiscriminate increases or decreases in expression of all genes in the signature.

CD4 T cells were chosen for these experiments because of the availability of reagents for magnetic isolation of large numbers of naive or memory-phenotype cells. It is important to note that for these proof of principle experiments, our test populations of lymphocytes were *memory-phenotype *cells, rather than those with known functional properties of memory lymphocytes. Unequivocally identifying human T cells with the functional properties of memory – such as antigen-independent survival and the ability to self-renew – is not feasible; instead we chose a to study cells with a well recognized memory-phenotype as this population is presumably enriched for those cells that have memory function. The method of purifying populations of naive and memory-phenotype cells did not influence the expression levels in the signature, and comparison of results from cells flow-sorted or purified with MACS were highly correlated (data not shown). However, MACS purification allowed more rapid isolation of the number of cells needed for screening applications.

### Quantitation of Differentiation Signature Genes in T Cells

Gene-specific primers were designed for each of the 55 genes in the differentiation signatures and for four control genes with which to normalize expression level data between replicate wells. Control genes were chosen (*ACTB*, *TUBB*, *TUBG1*, and *HNRPAB*) that showed low signal-to-noise ratios of expression level in Affymetrix data between naive and memory-phenotype CD4 T cells, and which spanned the range of expression levels seen in the transcripts of the differentiation signature. In subsequent analyses each gene in the differentiation signature was normalized to the mean of these four genes.

**Figure 3:**
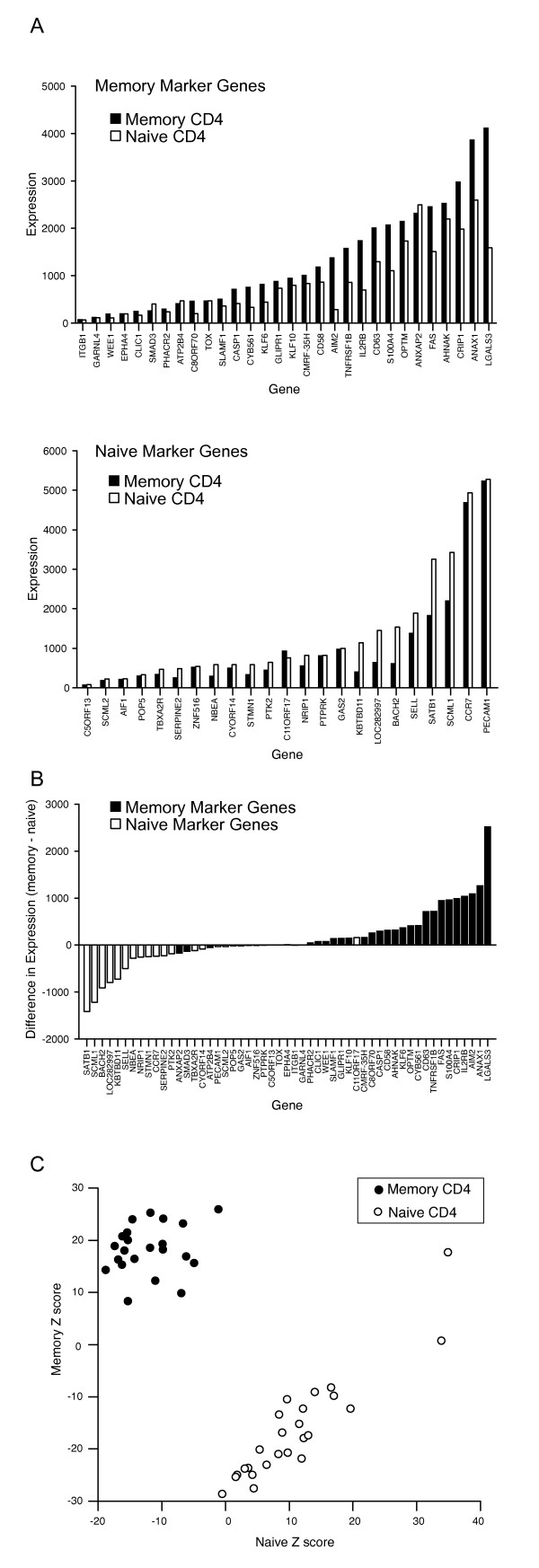
**Amplification of differentiation signature genes in primary human T cells**. (A) Expression level (aribrary units) of memory-phenotype marker genes (upper panel) or naive marker genes (lower panel) measured in sorted populations of naive (white bars) or memory-phenotype (black bars) peripheral blood CD4 T cells (25,000 cells/well). Each bar represents mean of ~6 replicate wells. Standard deviation was < 8% (not shown) in all cases. (B) Difference in expression level for naive (white bars) or memory-phenotype (black bars) marker genes between memory-phenotype and naive CD4 T cells. Values > 0 show relative increase in expression in memory-phenotype CD4 T cells; < 0 show relative increase in naive CD4 T cells. (C) Cumulative score of memory-phenotype (plotted on Y-axis) or naive marker genes (X-axis) in effector/memory phenotype (black symbols) or naive (white symbols) CD4 T cells. Values expressed as Z scores to compensate for wide variation in expression level of individual genes.

Naive and memory-phenotype CD4 cells were flow-sorted using the gating strategy shown in Figure 2 and plated at various cell densities per well in 96-well U-bottom plates. Following cell lysis, LMA and bead-detection were performed (Fig 3). All 59 genes were successfully amplified, indicating that the method can reliably amplify a large number of genes simultaneously. Cell titration experiments demonstrated that optimal gene expression data were obtained at cell numbers of 25,000 per well or above (data not shown); all subsequent experiments were performed using 25,000 cells/well. The absolute transcript expression values spanned a 3-log range (Fig. 3A). The most abundant transcripts in the memory-phenotype differentiation signature included LGALS3 and FAS, and in the naive differentiation signature, CCR7 and PECAM1. However, even transcripts that would be expected to be expressed at a low copy number such as the transcription factors KLF10 or BACH2 were amplified reliably suggesting efficient amplification of even low abundance transcripts.

Comparison of expression levels of naive or memory genes in naive and memory-phenotype cells (Fig. 3B) showed that the difference in expression level between naive and memory-phenotype cells was large for some genes, such as LGALS3, ANAX1 (upregulated in memory-phenotype compared to naive), and SATB1 and SCML1 (upregulated in naive compared with memory-phenotype). However, for other genes relatively small differences in expression between each cell type were observed (e.g. KLF10 or SERPINE2). The magnitude of difference in gene expression was not related to the overall transcript abundance: genes such as AHNAK were expressed at high levels but demonstrated small fold-difference in expression between effector/memory phenotype and naive cells. However, while the difference in expression level for individual transcripts was small for many of the genes in the signature, the overall direction of change in expression levels was as predicted for the majority of memory-phenotype marker genes and naive marker genes (Fig 3B), i.e. the vast majority of genes expected to be higher in memory-phenotype cells were indeed expressed at higher levels than in naive cells and vice-versa. Overall ~10% of transcripts in the panel were "contrarian", or expressed at relative levels opposite to that predicted (e.g. ANXAP2, SMA3). Such genes were not included in subsequent analyses.

We reasoned that measuring multiple genes representative of a cell state would provide a more robust discrimination of cell state than relying on a single gene because small but consistent differences in expression level of signature genes could be aggregated. To test this, the expression level of the differentiation signature genes were measured in multiple replicates of memory-phenotype and naive CD4 T cells (Fig 3C). For each well, the expression of memory-phenotype marker genes or naive marker genes were summed (after conversion to a Z-scores to compensate for the wide range in absolute expression values) and expressed as two vectors or "scores" on a bivariate plot. The cumulative scores of expression values for memory-phenotype or naive marker genes separate memory-phenotype (black symbols) or naive (white symbols) CD4 T cells more clearly than does the expression level of any of the individual transcripts.

To quantify this difference, Z' factors were calculated for individual genes and for the summary score of expression values of all genes. The Z' factor is used to evaluate the ability of assays to detect true positive results in high-throughput screens [17]. The theoretical maximum value is 1, and values > 0.5 suggest excellent ability to detect true positive 'hits'. In contrast, values below 0 do not allow discrimination of positive and negative results. As shown in Table 1, the Z' factor for this signature was 0.68, and the highest score was achieved by aggregating all genes in the differentiation signature. No single gene provided as high resolution, and only three individual genes (*S100A4*, *LGALS3*, and *ANAX1*) gave Z' factors above 0. Thus the use of a signature-based assay was superior to any single marker gene in distinguishing naive and memory-phenotype CD4 T cells.

**Table 1:** Z' Factor scores for the aggregate genes in the signature (summary score) or for the highest scoring three individual genes.

**Parameter**	**Z' Factor**
Weighted summary score (all genes)	0.65
S100A4	0.45
LGALS3	0.36
ANAX1	0.3
All other genes individually	<0

### Comparison of LMA with Affymetrix gene expression profiling

**Figure 4:**
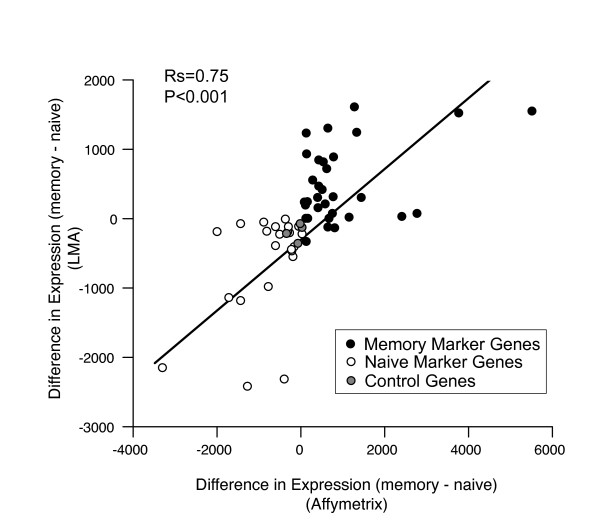
**Comparison of gene expression levels of differentiation signature genes measured by different platforms**. Difference in expression level of naive (white symbols) or memory-phenotype (black symbols) marker genes or control genes (grey symbols) between memory-phenotype and naive CD4 T cells. Values > 0 show relative increase in expression in memory-phenotype CD4 T cells; < 0 show relative increase in naive CD4 T cells. Expression levels for corresponding genes measured by LMA are plotted on Y axis; by Affymetrix U133A microarray on X-axis. Each point represents the mean values from cells sorted from ~4 – 6 subjects. R*s *and *P *value refer to Spearman correlation coefficient.

We next considered how well the method recapitulated the changes in gene expression measured by Affymetrix microarrays. We compared the difference in gene expression levels between memory-phenotype and naive CD4 T cells measured by LMA/bead-based detection with the original Affymetrix microarray data shown in Figure 1. The values were highly correlated between platforms (Fig 4). This is especially striking because samples from different healthy donors were used to generate the expression data on each platform. Thus the changes detected by LMA/bead-based detection are similar to those measured by Affymetrix microarray.

### Differentiation signature is comparable between donors

**Figure 5:**
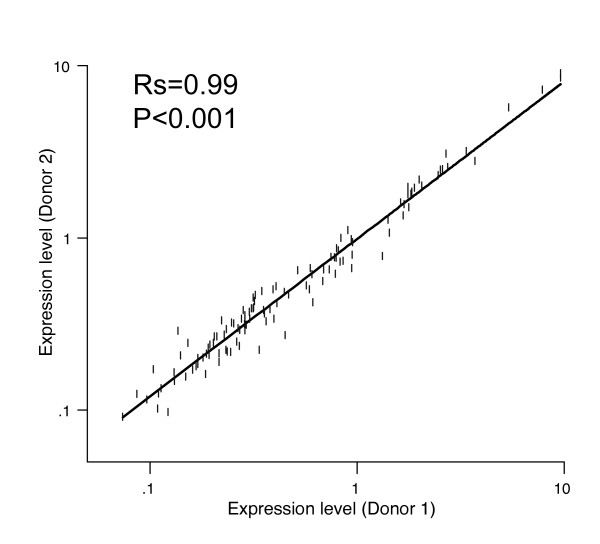
**Minimal donor variation in relative expression of differentiation signature in different donors**. Correlation of expression level for each transcript in naive or memory-phenotype CD4 T cells measured by the method in two different healthy donors. Rs and P value refer to Spearman correlation coefficient.

We next evaluated the variability of the gene expression level detection across multiple replicates of naive and memory-phenotype CD4 T cells from two donors. Being able to perform screens involving large numbers of compounds would require pooling data from multiple donors. To identify the extent of donor-to-donor variability, we compared the expression levels of all genes in naive and memory-phenotype CD4 T cells in two donors (Fig 5). The expression levels were highly correlated (Rs = 0.99), suggesting that there is little variability in the relative expression levels of this set of transcripts between populations of phenotypically similar CD4 T cells in the two donors studied.

### Assay is precise and robust

To function as a screening tool that can be scaled up to high-throughput, the assay should ideally be robust and precise, even when used at high-throughput with robotic fluid handling. We therefore performed the assay in large numbers of replications (~100 per cell type) in CD4 T cells obtained from a leukapheresis product from a healthy donor.

**Figure 6:**
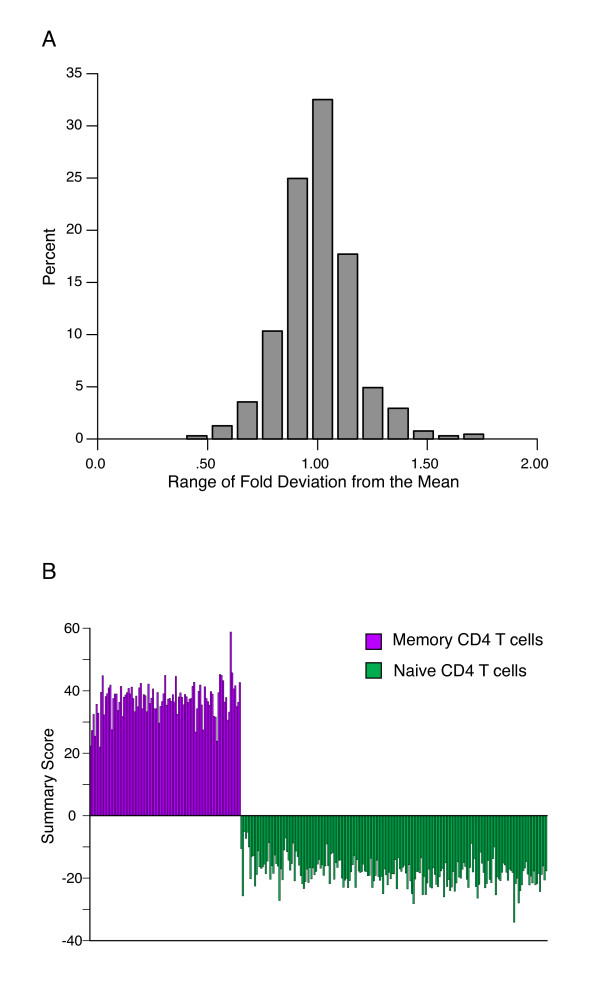
**Evaluation of LMA/Luminex detection assay reproducibility and scalability**. (A) Mean expression level for each transcript in naive or memory-phenotype CD4 peripheral blood T cells were calculated and the deviation of that data point from its corresponding mean were calculated. The fraction of data points in each of 12 bins of fold deviation values is shown, representing 1100 data points (two differentiation states × 55 transcripts × 6 replicates). (B) Cumulative Z-score for multiple replicates of memory-phenotype (purple) or naive (green) CD4 T cells measured by the method carried out using robotic automation. Values > 0 show relative increase in expression of memory-phenotype signature genes; < 0 show relative increase of naive signature genes.

After MACS purification of naive and memory-phenotype CD4 T cells, cells were plated into a 384 well plate. The cells were incubated for 18 hours (to simulate exposure to test compounds), followed by harvest, LMA, and bead detection using robotic fluid handling (Fig 6). Replicate measurements of genes in the differentiation signature were highly correlated, and all data points fell within two-fold of their corresponding means (Fig 6A). The Z' Factor for the memory-phenotype versus naive comparisons in scaled up and automated experiments were ≥ 0.5, suggesting that the assay can be performed on a scale necessary to permit high-throughput screens.

## Discussion

The ability to identify compounds that direct the cellular differentiation of naive T cells into memory cells could have significant therapeutic impact. However, the lack of reporter assays suitable for high-throughput that can accurately distinguish between discrete differentiation states in T cell development has limited this approach. Here we describe the application of a gene expression-based assay that detects complex, multigene signatures and distinguishes samples of naive and memory-phenotype CD4 T cells from human peripheral blood at high-throughput.

Traditionally, reporter gene assays measure the transcriptional activity of a single gene that is characteristic of the differentiated state. However, in the case of memory lymphocyte differentiation, no single gene can accurately distinguish between naive and memory-phenotype cells. For instance, the naive markers CCR7 and CD62L are both re-expressed by central memory cells preventing these genes in isolation from being used to separate the two cellular states [18]. Similarly, genes that are not expressed by naive T cells such as granzyme B or perforin are expressed by both effector and memory-phenotype cells making it difficult to screen for compounds that direct differentiation only towards a memory state [11]. We took the approach of identifying a signature of genes that are differentially expressed during memory differentiation both in humans and mice, regardless of their functional role. This cross-species signature readily discriminated between the test populations of cells in these experiments – phenotypically naive cells that would not be expected to manifest the memory differentiation signature, and memory-phenotype cells that, as a population, would be expected to show increased expression of a memory differentiation signature. The assay that we used simultaneously measured the expression level of a panel of 55 genes that included transcripts increased in memory-phenotype CD4 T cells compared to their naive counterparts and an equivalent number that show the opposite profile. We found that, as expected, the expression level of each gene in isolation was often not markedly different between naive and memory-phenotype CD4 T cells. However, most memory-phenotype marker genes were consistently expressed at higher levels in memory-phenotype cells than in naive cells; similarly, naive-phenotype genes were consistently expressed in greater abundance in naive CD4 T cells than in memory-phenotype cells. When the resulting, albeit small, difference in expression level of each of a gene in the panel was aggregated, naive and memory-phenotype cells could be easily distinguished with a Z' factor score better than any gene in isolation. This demonstrates that a signature-based screening platform can provide a powerful degree of resolution in distinguishing differentiation states that lack clear-cut differentiation markers.

We developed this assay to allow screening to be done using primary human lymphocytes. The use of primary human cells as starting material is highly unusual for differentiation-based screens but offers significant advantages over cell lines. The analysis of primary cells *ex vivo* more closely recapitulates the cellular state *in vivo* than does the analysis of cell lines. Moreover, primary cells may be more sensitive to toxicities of compounds identified within a screen allowing earlier detection of potentially harmful drugs. Three factors suggest that using primary human T cells for memory differentiation screens will prove feasible. First, T cells are relatively abundant and can be accessed in normal volunteers with minimal morbidity. Second, selection of subsets within the peripheral T cell compartment is technically straightforward with a number of available cell separation techniques. Third, we found a remarkable degree of consistency in the relative expression level of genes in the differentiation signature between two different donors. This fact is of great practical significance as several different donors would be needed to contribute naive T cells to a screen for a high-complexity compound library. Further evaluation across a larger number of normal donors will be necessary to define rigorously how significant an obstacle donor-to-donor variability in gene-expression will be in the conduct of large screens spanning more than one donor. However, the lack of donor-to-donor variability in these initial studies suggests that analysis of pooled screening data from multiple donors may be possible.

There are clearly caveats associated with a signature-based approach to HTS in primary cells. The signature of genes was defined empirically, and so the biological relevance of any of the genes in the signature to the differentiation states of interest is unknown. However, the differentiation signature used in this study is highly evolutionarily conserved, shared by memory differentiation in both T and B cell lineages, and disrupted in functionally exhausted T cells [12]. This suggests that this gene signature *in toto *is highly correlated with the differentation state of functional memory cells. Never-the-less, future studies will be required to determine whether it is possible to elicit the complex gene expression profile representative of a given differentiation state without inducing its attendant functional properties. Second, the use of primary human cells is technically challenging. Like all primary cells we found that increasing time in culture altered the gene expression profiles, presumably due to the lack of input signals from the normal milieu found *in vivo* (data not shown). However, data from mouse models suggests that the lineage commitment step for naive T cells differentiating after antigen-encounter occurs within hours or days of initial TCR signaling [19-22]. This suggests that a brief exposure to compound may be sufficient to direct differentiation of naive T cells to a memory fate before adverse *ex vivo* effects are apparent in the differentiation signature.

Our data suggests that it is now possible to screen for compounds that induce memory differentiation in naive human T cells. Compounds identified in such a screen would have significant biological and therapeutic uses. First, as tool compounds, hits in a memory differentiation screen could afford new insights into the mechanisms underlying memory development. Second, as therapeutic agents, memory-differentiating drugs could be used to accelerate the *ex-vivo* expansion of antigen-specific T cells or improve the efficiency of current vaccination approaches. Although widescale differentiation of naive T cells into memory cells would be undesirable in a clinical situation, antigen-specificity on promoting memory differentiation could be achieved by co-administration with vaccines, by optimization of dose and/or timing, or by the use of *ex-vivo* treatment of antigen-specific T cells.

## Conclusion

The use of LMA with bead-based detection offers a generic solution for T cell differentiation-based screens where no single-gene or functional assay is available. The same experimental platform can be easily adapted to other screening applications by using a panel of genes specific to the biology of interest. For instance, screens to detect the differentiation of effector memory T cells into those with a central memory gene expression signature could be designed based on their different expression profiles. Screens to convert conventional CD4 T cells into regulatory T cells could be developed by using a transcriptional profile of genes differentially expressed between conventional CD4 T cells and Tregs [23,24]. Similarly, screens to identify compounds that differentiate exhausted CD8 T cells into functional T cells can be designed using a panel of genes representing each state [25]. Assaying primary human T cells or mouse cells can be accommodated by the design of species-specific probes corresponding to orthologous genes. The ability to perform differentiation screens in T cell immunology with previously "unscreenable" phenotypes, and without the need to develop entirely new screening assays each time could significantly accelerate the discovery of compounds, genes or soluble factors that influence the development of T cell immunity in humans.

## Methods

### Research Subjects and cell purification

Peripheral blood samples were obtained from healthy volunteers who gave informed consent for research and were enrolled on a protocol approved by the DFCI IRB. Written informed consent was obtained from the subjects for publication of data in this report. A copy of the written consent is available for review by Editor-in-Chief of this journal. Peripheral blood mononuclear cells were purified by density centrifugation. Naive or effector/memory phenotype CD4 T cells were obtained either by negative selection over a magnetic column (Naive or Memory CD4+ T cell Isolation Kit, Miltenyi Biotec) or by flow sorting. For sorting, cells were stained with a cocktail of antibodies designed to exclude irrelevant lineages, Annexin V to exclude dead/dying cells, and CD4, CD45RA and CD62L to identify memory-phenotype and naive CD4 T cells as shown in Figure 2. Purity of MACS isolated naive cells was routinely > 95%, and sorted cells was > 99%.

### Ligation-mediated amplification

Purified populations of naive or memory-phenotype cells were plated in 384-well tissue-culture plates at 25,000 cells per well in 50 uL of RPMI supplemented with 10% human AB-serum (Valley Biomedical, Inc.) and antibiotics, and incubated at 37°C with 5% CO_2 _for ~18 hours. After incubation, 40 uL of medium was removed from the wells with a Multimek robot (Beckman Coulter). Cells were lysed with the addition of 10 ul of TCL lysis buffer (Qiagen) for 20 minutes at room temperature. The lysate was transferred to a 384-well oligo-dT coated plated (TurboCapture 384 mRNA kit, Qiagen) and incubated for 1 hour at room temperature. Excess lysate was removed by briefly centrifuging inverted uncovered plates onto absorbent paper towel. Reverse transcription was carried out in a 5 uL reaction volume using MMLV reverse transcriptase at 37°C (Promega). After a 90 minute incubation, liquid was removed from the wells by inverted centrifugation of the plates. Gene-specific oligonucleotide probes (Additional file 1) were hybridized to the plate-bound cDNA using 2 nM of each probes (118 probes in total). Upstream probes were designed to contain a T7 primer sequence, Luminex-designed FlexMap barcode tag (24 nucleotides in length) and the gene-specific sequence (20 nt). Downstream probes were phosphorylated at the 5' end to allow subsequent DNA ligation, and contained gene-specific sequence followed by a T3 primer sequence. Gene-specific probes were designed such that the upstream and downstream probes were of similar G-C composition, minimal repeats, and abutted at C and G or G and C nucleotides. Probe hybridization was performed at 95°C for 2 minutes followed by 50°C for 1 hour. Excess probe was removed by inverted centrifugation of the plate. DNA ligation step was then performed using Taq ligase (New England Biolabs) in a 5 uL volume at 45°C for 1 h followed by incubation at 65°C for 10 minutes. Excess ligation mix was spun out by inverted centrifugation of the plate. The ligated products were amplified with a universal, biotinylated T3 primer (5'-ATT AAC CCT CAC TAA AGG GA-3') and a universal T7 primer (5'-TAA TAC GAC TCA CTA TAG GG-3') using HotStarTaq DNA polymerase (Qiagen) in a 5 uL reaction volume.

Amplicon detection was carried out using xMAP Multi-Analyze COOH microspheres (Luminex, 2.5 × 10^6^) coupled to FlexMap barcode sequences that were complementary to barcode sequences contained in the gene-specific probe pairs. Coupling reactions were performed as previously described [14]. A 5 uL aliquot of the ligation-mediated amplification mix was hybridized to a mixture of microspheres containing ~2500 fluorescently distinguishable microspheres per gene in 18 uL of 1.5× TMAC (4.5 M tetramethylammonium chloride, 0.15% N-lauryl sarcosine, 75 mM Tris-HCl [pH 8], and 6 mM EDTA [pH 8]) and 5 uL of TE [pH8] at 95°C for 2 min and then 45°C for 1 h. To detect the amplicon bound to each bead, the sample was incubated with 20 ul of streptavidin-PE (Invitrogen) in 1× TMAC for 5 min at 45°C, washed twice, and resuspended in 1× TMAC. Dual-color fluorescence was detected with a Luminex 200, or high-throughput detection instruments. A minimum of 30 events was recorded per microsphere, and the median intensity on the PE channel recorded per bead.

### Data analysis

The median fluorescence intensity of PE corresponding to a gene-specific bead was used as a measure of the gene's expression level. Expression levels of each signature gene in the well were indexed to a mean of 4 control genes (*ACTB, TUBG, TUBB *and *HNRAPB*) in the corresponding well to minimize well-to-well variability. Filtering was performed to eliminate wells with aberrant control gene expression from further analysis, using the mean plus or minus two times the standard deviation of the control genes as a filtering threshold. Aggregate scores of all genes in the panel created a summed score of the indexed expression values of the memory and naive genes with a sign determined by the expected direction of expression in the two differentiation states. For some visualizations, normalization of the gene expression values were performed using a Z-score derived by subtracting the mean expression level of the gene in that class from a the raw gene expression value in a given well and then dividing the difference by the standard deviation of the gene's expression in that class.

## Authors' contributions

WNH conceived and designed the experiments, analyzed the data and wrote the manuscript. JA, KB, KR, CH, and SN performed the experiments and acquired the data. KR analyzed the data. KS helped design the experiments, analyze the data and write the manuscript.

## Supplementary Material

Additional file 1 Supplementary Table 1. Sequence information for gene-specific probes. Click here for file
