# Supplementary material for: High-throughput gene expression profiling of memory differentiation in primary human T cells
Source: BMC Immunol. 2008 Aug 1;9:44. doi: 10.1186/1471-2172-9-44 (PMC2529265; doi:10.1186/1471-2172-9-44)
Supplement: Additional file 1 — Supplementary Table 1. Sequence information for gene-specific probes. [file 1471-2172-9-44-S1.doc]

**Supplementary Table 1.** Sequence information for gene-specific probes.

| **Type** | **Gene** | **Tag#** | **Upstream Probe** | **Downstream Probe** |
| --- | --- | --- | --- | --- |
| Naïve | **KBTBD11** | 1 | TAATACGACTCACTATAGGGCTTTAATCTCAATCAATACAAATC AATCCTGGGCATAAATAGTG | CAGAGCCTCGTATGTTTGTCTCCCTTTAGTGAGGGTTAAT |
| Naïve | **SERPINE2** | 2 | TAATACGACTCACTATAGGGCTTTATCAATACATACTACAATCATAGACAAGGTTGCTGTTGTG | CAGTGTGCCTGTCACTACTGTCCCTTTAGTGAGGGTTAAT |
| Naïve | **LOC282997** | 3 | TAATACGACTCACTATAGGGTACACTTTATCAAATCTTACAATCTTCCTGTAAGAACTTCAGCC | CCTCTCAGGACCTTTTTCTT TCCCTTTAGTGAGGGTTAAT |
| Naïve | **C11ORF17** | 4 | TAATACGACTCACTATAGGGTACATTACCAATAATCTTCAAATC AACTTGAGGGTGTAGAGGTC | CTCCACGCTTGTTTGCCTGATCCCTTTAGTGAGGGTTAAT |
| Naïve | **CCR7** | 5 | TAATACGACTCACTATAGGGCAATTCAAATCACAATAATCAATCCTTTCGATTCGTTAAGAGAG | CAACATTTTACCCACACACATCCCTTTAGTGAGGGTTAAT |
| Naïve | **BACH2** | 6 | TAATACGACTCACTATAGGGTCAACAATCTTTTACAATCAAATCCTCTGTACCTCTCATAACTG | GTCAACGACTGTAACAGGTTTCCCTTTAGTGAGGGTTAAT |
| Naïve | **C5ORF13** | 7 | TAATACGACTCACTATAGGGCAATTCATTTACCAATTTACCAAT TGGATAAGATTGCTTCCTCC | CTCTCCTCATTACGTGTTCTTCCCTTTAGTGAGGGTTAAT |
| Naïve | **PECAM1** | 8 | TAATACGACTCACTATAGGGAATCCTTTTACATTCATTACTTACTTGTAGTGAGCGGAGATCGC | GCCACTGCACTCCAGCCTGATCCCTTTAGTGAGGGTTAAT |
| Naïve | **POP5** | 9 | TAATACGACTCACTATAGGGTAATCTTCTATATCAACATCTTAC GAACAGAACAATCTGGGTA | CAACAGCATCTTCCACAGTTTCCCTTTAGTGAGGGTTAAT |
| Naïve | **SCML1** | 10 | TAATACGACTCACTATAGGGATCATACATACATACAAATCTACA TAGATTGGTTTCCATACAGG | GAAGTTCTCCGTCCTATGCATCCCTTTAGTGAGGGTTAAT |
| Naïve | **GAS2** | 11 | TAATACGACTCACTATAGGGTACAAATCATCAATCACTTTAATC ATGAGTTATTTCACACATTC | CTGAGCACATGGCTGTGTTTTCCCTTTAGTGAGGGTTAAT |
| Naïve | **SATB1** | 12 | TAATACGACTCACTATAGGGTACACTTTCTTTCTTTCTTTCTTTCTTGGCAGCCCCAGGTGAAG | CATCAAGGATTGTTTGGTATTCCCTTTAGTGAGGGTTAAT |
| Naïve | **PTK2** | 15 | TAATACGACTCACTATAGGGATACTTCATTCATTCATCAATTCA ATATATGGACATGGCAGGCC | GATTTGGGAACCAAGCTATTTCCCTTTAGTGAGGGTTAAT |
| Naïve | **AIF1** | 16 | TAATACGACTCACTATAGGGAATCAATCTTCATTCAAATCATCATCTACACGTTGCCCATCATC | CCTTCTTCCATCCTTAGAGGTCCCTTTAGTGAGGGTTAAT |
| Naïve | **NBEA** | 18 | TAATACGACTCACTATAGGGTCAAAATCTCAAATACTCAAATCAGTACATATTGTGTCGGTAGG | GCTATGAGGCATGTTACAGGTCCCTTTAGTGAGGGTTAAT |
| Naïve | **ZNF516** | 19 | TAATACGACTCACTATAGGGTCAATCAATTACTTACTCAAATACTGTGTGTTGGACAGAGTGGC | GAGGCTGAGCACTGTCACAGTCCCTTTAGTGAGGGTTAAT |
| Naïve | **NRIP1** | 20 | TAATACGACTCACTATAGGGCTTTTACAATACTTCAATACAATCTGCATGAAAAGTGGCATGGG | GGACCCTGTGCATCTGTGCATCCCTTTAGTGAGGGTTAAT |
| Naïve | **PTPRK** | 21 | TAATACGACTCACTATAGGGAATCCTTTCTTTAATCTCAAATCATTTTTCAGCCTGTGGCCCAG | CACTGGTCAAGAAAACAAGATCCCTTTAGTGAGGGTTAAT |
| Naïve | **TBXA2R** | 22 | TAATACGACTCACTATAGGGAATCCTTTTTACTCAATTCAATCAGGAAGGCTCTGTCCAGAAAG | GATTGAATGTGAAACGGGGGTCCCTTTAGTGAGGGTTAAT |
| Naïve | **SCML2** | 23 | TAATACGACTCACTATAGGGTTCAATCATTCAAATCTCAACTTTGCACTTTGCACTGCCACAGG | GGTGACGATGGAACTATGTATCCCTTTAGTGAGGGTTAAT |
| Naïve | **STMN1** | 24 | TAATACGACTCACTATAGGGTCAATTACCTTTTCAATACAATACGTCTCCTTCCACAATGACTG | CTTTGTTTGGATGCCTCAGCTCCCTTTAGTGAGGGTTAAT |
| Naïve | **SELL** | 25 | TAATACGACTCACTATAGGGCTTTTCAATTACTTCAAATCTTCACACCTCTCTTTTTCAGTTGG | CTGACTTCCACACCTAGCATTCCCTTTAGTGAGGGTTAAT |
| Naïve | **CYORF14** | 26 | TAATACGACTCACTATAGGGTTACTCAAAATCTACACTTTTTCATCTGCCGCATCTTGATACTG | GACTTCCAGTCTCCAGAACTTCCCTTTAGTGAGGGTTAAT |
| Control | **TUBB** | 61 | TAATACGACTCACTATAGGGAATCTTACCAATTCATAATCTTCACCCAAAAAAGAATGAACACC | CCTGACTCTGGAGTGGTGTATCCCTTTAGTGAGGGTTAAT |
| Control | **ACTB** | 62 | TAATACGACTCACTATAGGGTCAATCATAATCTCATAATCCAATGTCATTCCAAATATGAGATG | CATTGTTACAGGAAGTCCCTTCCCTTTAGTGAGGGTTAAT |
| Control | **TUBG1** | 63 | TAATACGACTCACTATAGGGCTACTTCATATACTTTATACTACATCAGAGCACAGATCAGGGAC | CTCACGCATCTCTTTCTCATTCCCTTTAGTGAGGGTTAAT |
| Control | **HNRPAB** | 64 | TAATACGACTCACTATAGGGCTACATATTCAAATTACTACTTACGCCTGGACCTGTGGACCCTG | GTTGTAAAGAGTAAATTGTATCCCTTTAGTGAGGGTTAAT |
| Memory | **ADAM19** | 27 | TAATACGACTCACTATAGGGCTTTTCAAATCAATACTCAACTTTTTGTGAAGAGCAAGGAAGTG | CATTAGTAGAAGCCACCTGGTCCCTTTAGTGAGGGTTAAT |
| Memory | **C1ORF24** | 28 | TAATACGACTCACTATAGGGCTACAAACAAACAAACATTATCAAGTGATCTGTGGGACTGTCTG | GGCCTGTTACTCATCCTGCT TCCCTTTAGTGAGGGTTAAT |
| Memory | **ANXA1** | 29 | TAATACGACTCACTATAGGGAATCTTACTACAAATCCTTTCTTTCTCACAGCTATCGTGAAGTG | CGCCACAAGCAAACCAGCTTTCCCTTTAGTGAGGGTTAAT |
| Memory | **TNFRSF1B** | 30 | TAATACGACTCACTATAGGGTTACCTTTATACCTTTCTTTTTACTGGAGACAAGAAGGGTTTTC | CACCCTGGAATCAAGATGTATCCCTTTAGTGAGGGTTAAT |
| Memory | **CRIP1** | 31 | TAATACGACTCACTATAGGGTTCACTTTTCAATCAACTTTAATCGGAGACCCCATCCTTGGCTG | CTTGCAGGGCCACTGTCCAGTCCCTTTAGTGAGGGTTAAT |
| Memory | **IL2RB** | 32 | TAATACGACTCACTATAGGGATTATTCACTTCAAACTAATCTACCCTCTCTGCAAGTCGGTCTC | CTTATCCCCCCAAATGGAAATCCCTTTAGTGAGGGTTAAT |
| Memory | **GLIPR1** | 33 | TAATACGACTCACTATAGGGTCAATTACTTCACTTTAATCCTTTACAGCGAGACCAAGTGAAAC | GTTACTACTCTGTTGTATATTCCCTTTAGTGAGGGTTAAT |
| Memory | **SMAD3** | 34 | TAATACGACTCACTATAGGGTCATTCATATACATACCAATTCATATCAAGTATGGTAGGGGAGG | GCAGGCTTGGGGAAAATGGCTCCCTTTAGTGAGGGTTAAT |
| Memory | **KLF6** | 35 | TAATACGACTCACTATAGGGCAATTTCATCATTCATTCATTTCA ACTGAGAATGCTAATGGTTG | GGTTGATTGTATGTTGAGGATCCCTTTAGTGAGGGTTAAT |
| Memory | **CLIC1** | 36 | TAATACGACTCACTATAGGGCAATTCATTTCATTCACAATCAATGGGGATGAGGGAAAGAAATG | GGGGGCCTGGGTCAGATTTT TCCCTTTAGTGAGGGTTAAT |
| Memory | **GARNL4** | 37 | TAATACGACTCACTATAGGGCTTTTCATCTTTTCATCTTTCAATACTGTGGCTTGGAAGTGATG | CTCCATGTGACGACGACTTTTCCCTTTAGTGAGGGTTAAT |
| Memory | **SLAMF1** | 38 | TAATACGACTCACTATAGGGTCAATCATTACACTTTTCAACAATGAAGAAGAGGTAAAACGAAC | CATTACCAGACAACAGTGGATCCCTTTAGTGAGGGTTAAT |
| Memory | **CASP1** | 40 | TAATACGACTCACTATAGGGCTTTCTACATTATTCACAACATTA ACTGTATGAATGTCTGTGGG | CAGGAAGTGAAGAGATCCTTTCCCTTTAGTGAGGGTTAAT |
| Memory | **PHACTR2** | 42 | TAATACGACTCACTATAGGGCTATCTTCATATTTCACTATAAACCACAAGACCTCTCTATAATG | GTAAATGTAAGACATCACCA TCCCTTTAGTGAGGGTTAAT |
| Memory | **CD58** | 43 | TAATACGACTCACTATAGGGCTTTCAATTACAATACTCATTACA ACAACCTGTATCCCAAGCAG | CGGTCATTCAAGACACAGATTCCCTTTAGTGAGGGTTAAT |
| Memory | **AHNAK** | 44 | TAATACGACTCACTATAGGGTCATTTACCAATCTTTCTTTATACCCAGCCAGTTTGGTGCTGAC | GGTGAGAGGAAATTAGAATCTCCCTTTAGTGAGGGTTAAT |
| Memory | **EPHA4** | 45 | TAATACGACTCACTATAGGGTCATTTCACAATTCAATTACTCAAGGCAAGAATTGGTATCACAG | CCATCACGCACCAGAATAAGTCCCTTTAGTGAGGGTTAAT |
| Memory | **CYB561** | 46 | TAATACGACTCACTATAGGGTACATCAACAATTCATTCAATACACTGAGTCACCATTTGGCTTC | GGCCTGGAAATAGTGTGTTATCCCTTTAGTGAGGGTTAAT |
| Memory | **ANXA2P2** | 47 | TAATACGACTCACTATAGGGCTTCTCATTAACTTACTTCATAATGAGCTGAGGCAGGAGAATGG | CTTGAACCCGGAAGGCAGAGTCCCTTTAGTGAGGGTTAAT |
| Memory | **KLF10** | 48 | TAATACGACTCACTATAGGGAAACAAACTTCACATCTCAATAATAGGAGTTTTTTGGGGATGTG | GAGGTAGTTGGGTAGAAAAA TCCCTTTAGTGAGGGTTAAT |
| Memory | **WEE1** | 49 | TAATACGACTCACTATAGGGTCATCAATCTTTCAATTTACTTACTAGGCATTGCATGAACCATG | GGATGATGATTCTGTGGAGGTCCCTTTAGTGAGGGTTAAT |
| Memory | **C8ORF70** | 50 | TAATACGACTCACTATAGGGCAATATACCAATATCATCATTTACTGCTGCTTGGACAGCTAGAG | CACATCCTCTAGTTAGTTTGTCCCTTTAGTGAGGGTTAAT |
| Memory | **AIM2** | 51 | TAATACGACTCACTATAGGGTCATTTCAATCAATCATCAACAATTGTAAGTTACCTGAAAGCTG | CAGTTCACAGGCTCCTCTCTTCCCTTTAGTGAGGGTTAAT |
| Memory | **OPTN** | 52 | TAATACGACTCACTATAGGGTCAATCATCTTTATACTTCACAATCAAGTGTGGAGAGGTTCTGC | CTGACATAGACACGTTACAGTCCCTTTAGTGAGGGTTAAT |
| Memory | **CD63** | 53 | TAATACGACTCACTATAGGGTAATTATACATCTCATCTTCTACACCTCCTCATCTGGGGGAGTG | GAATAGTATCCTCCAGGTTTTCCCTTTAGTGAGGGTTAAT |
| Memory | **S100A4** | 54 | TAATACGACTCACTATAGGGCTTTTTCAATCACTTTCAATTCATTGGACAGCAACAGGGACAAC | GAGGTGGACTTCCAAGAGTATCCCTTTAGTGAGGGTTAAT |
| Memory | **TOX** | 55 | TAATACGACTCACTATAGGGTATATACACTTCTCAATAACTAACGCAGCTTTGACTTTGACAGG | CGGTTTGTGCAGGAAAGCACTCCCTTTAGTGAGGGTTAAT |
| Memory | **FAS** | 56 | TAATACGACTCACTATAGGGCAATTTACTCATATACATCACTTTTAAACTGAAGCAGATACCTG | GAACCACCTAAAGAACTTCCTCCCTTTAGTGAGGGTTAAT |
| Memory | **ATP2B4** | 57 | TAATACGACTCACTATAGGGCAATATCATCATCTTTATCATTACAATGATGACAATCCTCTTGG | CATCACCCCACCCCACATTCTCCCTTTAGTGAGGGTTAAT |
| Memory | **ITGB1** | 58 | TAATACGACTCACTATAGGGCTACTAATTCATTAACATTACTACGCGTAAGGGATCTTCCTTGG | GATGACTTGATTGTGGGTGGTCCCTTTAGTGAGGGTTAAT |
| Memory | **LGALS3** | 59 | TAATACGACTCACTATAGGGTCATCAATCAATCTTTTTCACTTTGAATTTCTGGTGACATAGAC | CTCACCAGTGCTTCATATACTCCCTTTAGTGAGGGTTAAT |
| Memory | **CMRF-35H** | 60 | TAATACGACTCACTATAGGGAATCTACAAATCCAATAATCTCATTAGCTTTGTCAATCACAGCC | CCATAGGAACGTCTGGAATTTCCCTTTAGTGAGGGTTAAT |
